# Callous-Unemotional Traits Do Not Predict Functional Family Therapy Outcomes for Adolescents With Behavior Problems

**DOI:** 10.3389/fpsyg.2020.537706

**Published:** 2021-01-18

**Authors:** Dagfinn Mørkrid Thøgersen, Gunnar Bjørnebekk, Christoffer Scavenius, Mette Elmose

**Affiliations:** ^1^Department of Psychology, University of Southern Denmark, Odense, Denmark; ^2^Norwegian Center for Child Behavioral Development, Oslo, Norway; ^3^Department of Special Needs Education, University of Oslo, Oslo, Norway; ^4^VIVE – The Danish Center for Social Science Research, Copenhagen, Denmark

**Keywords:** callous-unemotional traits, adolescents, behavior problems, treatment, functional family therapy, family therapy

## Abstract

Despite the availability of evidence-based treatment models for adolescent behavior problems, little is known about the effectiveness of these programs for adolescents with callous-unemotional (CU) traits. Defined by lack of empathy, lack of guilt, flattened affect and lack of caring, CU traits have been linked to long-term anti-social behavior and unfavorable treatment outcomes and might be negatively related to outcomes in evidence-based programs such as Functional Family Therapy (FFT). This study used a single-group pre-post evaluation design with a sample of 407 adolescents (49.1% female, mean age = 14.4 years, *SD* = 1.9) receiving FFT to investigate whether outcomes in FFT are predicted by CU traits and to what extent reliable changes in CU traits can be observed. The results showed that although CU traits are related to increased problem severity at baseline, they predicted neither treatment dropout nor post-treatment externalizing behavior and family functioning. CU traits were related to diminished improvement ratings, in particular with respect to parental supervision. Reductions in CU traits were observed across the time of treatment, and these were most profound among adolescents with elevated levels of CU traits at baseline. Further research should investigate whether certain evidence-based treatment components are more suited for adolescents with CU, and if the addition of specific intervention elements for reducing CU-traits could further improve outcomes for this high-risk population.

## Introduction

Problem behaviors in adolescence can range in severity from heated verbal arguments and breaking curfew to aggression, violence, criminal behavior and drug use (Dishion and Patterson, 2006). These behaviors, especially if they started in early childhood, can have negative effects on family and peer relationships, academic achievement and predict mental health adversities, disturbed family life and economic hardship in adulthood (Colman et al., 2009; McLeod et al., 2012; Moffitt, 2015). Given its importance, decades of research in this field have informed the development of evidence-based treatment programs to curb adolescent behavior problems and diminish the risk for further anti-social development (McCart and Sheidow, 2016). This paper aims to increase our knowledge on whether adolescents with callous-unemotional (CU) traits can expect treatment gains in such evidence-based programs.

One of the most studied and widely implemented evidence-based programs for adolescent behavior problems is Functional Family Therapy (FFT; Alexander et al., 2013). FFT is an intensive, short-term (3–6 months) family-focused treatment where both the adolescent and his/her parent(s) participate in the therapy sessions. FFT comprises five distinct phases each with specific aims: (1) *Engagement* to ensure family members participation in sessions; (2) *Motivation* to decrease the level of blame and negativity in the family and increase hope and motivation for change; (3) *Relational Assessment* to assess how risk and protective factors and family relational dynamics relate to the behavior problems; (4) *Behavior Change* to improve family skills such as communication, conflict management and problem solving to reduce problem behavior; and (5) *Generalization* to maintain and extend skill use inside and outside the family, prevent relapse and refer the family to additional support and services, if needed. FFT has a primary focus on family level risk factors as the reduction of these have been linked to diminished behavior problems (Dishion and Patterson, 2006), and they are malleable through short-term psychosocial interventions stemming from behavioral, cognitive-behavioral and family systems orientations (McCart and Sheidow, 2016).

Meta-analyses of randomized controlled trials evaluating the efficacy of FFT find that it can reduce adolescent behavior problems in various samples and settings (Baldwin et al., 2012; Hartnett et al., 2017). However, there is variation in outcomes among adolescents who receive FFT (Sexton and Turner, 2010; Hartnett et al., 2017). Possible individual level moderators of FFT efficacy might include age, gender, symptom severity, problem duration and the presence or absence of additional risk factors and common co-morbidities, e.g., ADHD and anxiety (Barker et al., 2010; Polier et al., 2012). The current knowledge on such moderating factors of FFT is limited (Scavenius et al., 2019), and a critical individual risk factor that warrants particular attention among adolescents with behavior problems are CU traits.

CU traits closely resemble the affective dimension of adult psychopathy and are defined by four core elements: a lack of empathy, a lack of remorse and guilt, lack of concern about performance and shallow or deficient affect (Frick et al., 2014b). Under the term “with limited prosocial emotions,” CU traits have become a specifier for the Conduct Disorder (CD) diagnosis in DSM-V and ICD-11 (American Psychiatric Association, 2013; World Health Organization, 2018). Depending on the informant, CU traits are seen in 21–31% of clinic referred children with CD compared to 2–7% in a community sample of children without CD (Kahn et al., 2012). CU traits have been linked to increased and more persistent behavior problems, even when controlling for conduct problem severity, level of aggression, impulsivity, childhood Oppositional Defiant Disorder, and childhood onset of CD (Frick, 2012). The finding that CU traits at age 12 years is related to adult anti-sociality, suggests that these traits play an important role in the development of persistent criminal behavior (Goulter et al., 2019).

The link between CU traits and sustained anti-social behavior might be due to limitations in neuropsychological functioning. Several studies have demonstrated that children with CU traits have reduced affinity or sensitivity for fear stimuli as observed by reduced level of amygdala activation (Viding et al., 2012; Lozier et al., 2014), lack of attendance to negative emotions in others (Dadds et al., 2008; Fairchild et al., 2009; Hodsoll et al., 2014), reduced self-initiated eye gazing (Dadds et al., 2006), reduced face preference toward care-givers (Bedford et al., 2015) and abnormal processing of punishment ques (Frick et al., 2003). Diminished attention and reactivity to negative emotional stimuli might be particularly pertinent for adolescents with CU traits in combination with conduct problems (Szabó et al., 2019; Northam and Dadds, 2020). These limitations might interfere with the development of conscience and contribute to sustained anti-social behaviors (Frick et al., 2014b).

In addition, these neuropsychological impairments might serve as the basis for why children with CU traits respond differently to certain parenting practices. The lowered ability or propensity to be influenced by other’s distress could make children with CU traits less sensitive to harsh and coercive parenting, compared to children without CU traits (Frick et al., 2014a). Children with CU traits also seem to benefit less from the firm, but emotionally calm, rule and consequence-based parenting skills encompassed in evidence-based treatment programs for conduct problems, e.g., time-out strategies (Hawes and Dadds, 2005). Conversely, parental warmth seems to be an important protective factor in the development of CU traits in children (Kroneman et al., 2011; Pasalich et al., 2012; Fanti et al., 2017; Waller et al., 2018b; Ray et al., 2019). These differential effects are supported by research showing that only the positive and reward-focused elements of a parenting program benefited children with conduct problems and CU traits (Hawes and Dadds, 2005). In essence, children with CU traits seem more responsive to contextual influences through parental warmth and involvement compared to rule and consequence-based parenting and negative harsh parenting (Waller et al., 2013; O’Connor et al., 2016).

Based on this understanding, the context of FFT-treatment is interesting for studying whether CU traits predict observed changes in problem behaviors and whether CU traits are malleable. First of all, FFT consistently focus on engagement in therapy and increasing family member motivation, which might counter the low motivation for therapy related to CU traits (O’Neill et al., 2003). Secondly, FFT puts an emphasis on improving family relationships and interactions and reducing blame and negativity between family members. This focus on strengthening the parent-adolescent relationship and increasing parental warmth might be important protective factors for children with CU traits (Wilkinson et al., 2016). Thirdly, FFT places an emphasis on relational and strength-based interventions over consequence-based parenting strategies. This applies both for within-family relationships and toward school and peers, and is in line with the effectiveness of encouragement and rewards compared to negative consequences for children with CU traits (Hawes and Dadds, 2005). Finally, FFT individualizes treatment through analyses of behavioral patterns, risk factors and family dynamics, which should enable therapists to adjust interventions so that they match the needs of each adolescent and family (Alexander et al., 2013).

Still there is limited research on CU traits in the context of FFT. To our knowledge, only one study of 134 predominantly male juvenile justice involved adolescents has been conducted (White et al., 2013). The results showed that while CU traits were related to higher severity of conduct problems both prior to and after treatment, the association was weaker after treatment due to a larger decline in conduct problems for those with elevated CU traits. However, the relationship between CU traits at baseline and reductions in conduct problems, aggression and emotional symptoms was not statistically significant when controlling for baseline levels of the outcome measures (White et al., 2013). These results are in line with the lack of moderation effects for high levels of CU traits on outcomes observed for the Multisystemic Therapy program (Manders et al., 2013; Fonagy et al., 2018). However, as White et al. (2013) demonstrated, CU traits were related to both baseline and post-FFT problem severity, despite the similar levels of treatment gains. In addition, CU traits predicted lower self- and parent-reported treatment improvements and indicated a higher risk of violence during treatment (White et al., 2013). So, although adolescents with CU traits may obtain treatment gains in FFT similar to those without CU traits, they still remain a more at-risk group.

Furthermore, there is to our knowledge a lack of research on whether the CU traits change over the course of FFT treatment which could potentially reduce the future negative impact of this risk factor. Research on the Multisystemic Therapy program shows inconclusive results in relation to whether there are decreases in CU traits across treatment (Butler et al., 2011; Manders et al., 2013; Fonagy et al., 2018). These mixed findings and the lack of empirical investigation of the change in CU traits over the course of FFT-treatment, calls for further research using larger samples.

The aims of this study were therefore threefold. First, we sought to gain knowledge on how CU traits relate to clinical and demographic characteristics in a Danish at-risk sample. Based on previous research, we hypothesized that at baseline CU traits would be significantly related to higher levels of conduct problems, lower levels of anxiety, less prosocial behavior, gender (boys scoring higher than girls) and age (older scoring lower than younger) (Frick, 2012).

Secondly, we sought to evaluate if CU traits predict completion status and treatment outcomes in the context of FFT treatment. We assessed for this both related to adolescent behavior problems and family level problems. In line with previous research by White et al. (2013), we hypothesized that CU traits would neither increase nor diminish treatment gains when controlling for pre-treatment levels of the outcome measure and potential effects related to gender, age and treatment duration. Potential interaction effects between CU traits and gender were also explored.

Lastly, we wanted to determine the level of change in CU traits over time in the context of FFT-treatment. Previous research has shown mixed and limited evidence for the therapeutic malleability of CU traits in adolescents, so we hypothesized that for the majority of the participants in our study, CU traits would not reliably change and that any observed effect-size in relation to changes in CU traits would be small. We also examined the relationship between change in CU traits and change in adolescent behavior problems and family level problems.

## Materials and Methods

### Participants

**FIGURE 1:**
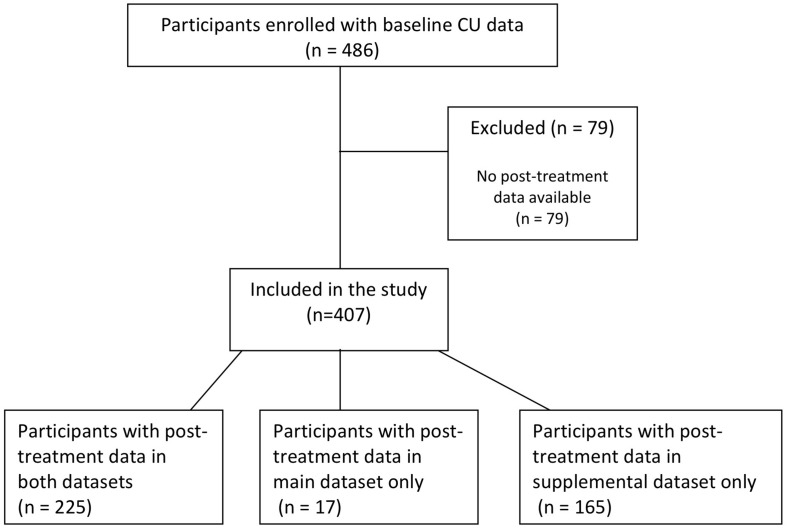
Participant data sources diagram.

This study builds upon data from a single-group pre-post design evaluation of FFT in eleven municipalities and two private treatment agencies in Denmark (Vardanian et al., 2019). Inclusion to the FFT intervention required that the municipality child welfare referred the family to family services and that a therapeutic team asserted FFT as the appropriate and available treatment for the family. The guidelines for referral to FFT specify inclusion criteria related to moderate to severe behavior problems (e.g., truancy, verbal aggression, violence, criminal behavior, and drug use) and exclusion criteria related to autism, suicidal behavior and psychosis. Adolescents were included in the current study based on (1) enrollment in the evaluation between September 2015 and March 2019, (2) data on CU traits at baseline was collected, and (3) post-treatment data from either the evaluation study or the FFT quality assurance system was available. Following these criteria, a total of 407 adolescents were included in the analyses, see Figure 1. The sample comprised of 207 (50.9%) boys and 200 (49.1%) girls with a mean age of 14.4 years (*SD* = 1.9). See Table 1 for additional characteristics of the sample by the main study variables.

**TABLE 1:** Descriptive statistics of the main study variables for the full sample.

	Baseline				Post-treatment			
	*n*	*M*	*SD*	Range	*n*	*M*	*SD*	Range
**ICU12**								
Parent-report	407	12.40	7.1	0–33	216	10.14	6.1	0–32
**SDQ-Externalizing**								
Parent-report	406	9.12	4.3	0–20	230	6.80	4.0	0–17
Self-report	376	9.00	3.8	0–20	213	7.71	3.7	0–19
**SDQ-emotional problems**								
Parent-report	405	4.77	2.6	0–10	230	3.37	2.4	0–10
Self-report	375	4.30	2.6	0–10	213	3.68	2.5	0–10
**SDQ-prosocial behavior**								
Parent-report	407	7.15	2.2	0–10	230	7.84	2.0	2–10
Self-report	377	7.49	2.0	0–10	213	7.79	1.7	2–10
**SCORE-15**								
Parent-report	405	2.52	0.72	1.0–4.8	225	2.10	0.58	1.0–3.9
Self-report	379	2.68	0.71	1.0–4.7	208	2.29	0.71	1.0–4.3

**ICU, inventory of callous unemotional traits; SCORE, systemic clinical outcome and routine evaluation; SDQ, strength and difficulties questionnaire*.*

### Measures

#### Callous Unemotional Traits

CU traits were measured by parent-reports on the 12-item Inventory of Callous-Unemotional traits (ICU12; Hawes et al., 2014). The ICU12 constitutes two intercorrelated factors, a 7-item Callousness factor where all items are standard-scored, e.g., “Shows no remorse when he/she has done something wrong,” and a 5-item Uncaring factor, where all items are reverse-scored, e.g., “Tries not to hurt others’ feelings.” Each item is rated on a 4-point scale, ranging from 0 (Not at all true) to 3 (Definitely true), and factor and total scores are the sum of the item responses. The ICU12 was developed using Item Response Theory analyses and research supports its use as a valid and brief measure of CU traits in both self- and parent-report versions (Hawes et al., 2014; Colins et al., 2015; Pechorro et al., 2017; Yoshida et al., 2019; Thøgersen et al., 2020). Reliability was good for the total scale (α = 0.91), and acceptable for the Callousness (α = 0.88) and Uncaring (α = 0.81) subscales. As the Callousness and Uncaring factors were highly correlated, *r*(405) = 0.689, *p* < 0.001, and the factor structure of the ICU12 might mostly be due to method variance effects (Cardinale and Marsh, 2017; Paiva-Salisbury et al., 2017; Ray and Frick, 2018; Thøgersen et al., 2020), only the total ICU12 score was used in this study. Norms for the 24-item version of the ICU suggests a clinical cut-off at 30 scale points for parent-report (Docherty et al., 2016). The mathematically equivalent cut-off score for a 12-item version is 15 scale points.

#### Behavioral and Emotional Problems

Adolescent behavioral and emotional problems were assessed by the self- and parent-report versions of the 25-item Strengths and Difficulties Questionnaire (SDQ; Goodman, 1997). Items on the SDQ are rated on a 3-point scale: 0 (Not True), 1 (Somewhat true) and 2 (Certainly True), and by five 5-item groups they make up the SDQ subscales: Prosocial behavior, Conduct Problems, Hyperactivity/Inattention, Emotional Symptoms and Peer relationship problems. In this study we used all but the peer relationship problems scale. The reliability was somewhat low for self- and parent-reports of conduct problems (α = 0.58 and α = 0.68, respectively), and also for self-reported prosocial behavior (α = 0.67) and parent-reported emotional problems (α = 0.69). All remaining subscales displayed acceptable reliability (α > 0.70). The lack of acceptable reliability for the Conduct problems subscale led to the choice of using the overarching Externalizing scale comprising the conduct problems and hyperactivity subscales as the primary outcome measure (α = 0.76 for self-report, α = 0.80 for parent-report).

#### Family Functioning

Family functioning was assessed by the 15-item Systemic Clinical Outcome and Routine Evaluation (SCORE-15; Carr and Stratton, 2017). In five item groups the items constitute three interrelated dimensions of family functioning: difficulties (e.g., “We seem to go from one crisis to another in my family”), communication (e.g., “People often don’t tell each other the truth in my family”) and strengths (reverse-scored, e.g., “We are good at finding new ways to deal with things that are difficult”). Each item is rated on a 5-point scale ranging from 1 (describes my family very well) to 5 (describes my family - not at all). The total score is an average of all 15 items with higher scores indicating poorer functioning. Prorating one missing item score per respondent is allowed when calculating the total score (Stratton et al., 2013). In an Irish sample of 139 school age adolescents the 90%-ile of the SCORE-15 using a 6-point scale, converts to 3.58 for a 5-point scale (Fay et al., 2013). The reliabilities of the SCORE-15 total scores were good for both adolescent-report (α = 0.91) and parent-report (α = 0.92) in our sample.

#### Client Reported Treatment Gains

The Client/Therapist Outcome Measure (COM/TOM) is a 6-item questionnaire completed at the end of FFT-treatment. Adolescents, parent(s) and therapist rate the family’s change on a general level (one item) and on five specific areas (one item per area): family communication skills, adolescent behavior, parenting skills, parental ability to supervise and monitor adolescent and family conflict levels. Each item is rated on a 6-point scale ranging from 0 (Things are worse) to 5 (Very much better), with a no change anchor at scale point 1 (Things are no different). Reliability across rater groups was good with αs ranging from 0.90 to 0.95.

#### Treatment Pacing, Duration and Completion

The FFT quality assurance database provided data on the number of days between each of the first three sessions (as a measure of initial treatment pacing), the number of sessions (as a measure of treatment intensity) and treatment duration. In addition, we collected the therapist assigned closing category, defined as either completion (family participated in all treatment phases and therapist assessed treatment as complete), drop out (family discontinued treatment prior to treatment completion), or non-completion (case closed prior to completion for reasons not related to therapy, e.g., administrative discharge due to the family moving).

### Procedures

The adolescents and their parents completed the study questionnaires prior to or during their first FFT sessions for the baseline assessment, and during their last FFT session or shortly thereafter for the post-treatment assessment. The time between baseline assessment and post-assessment ranged from 17 to 473 days, with a median of 169 days and interquartile range of 88 days. Therapists entered data for each case in the FFT quality assurance database in relation to session dates and treatment completion status.

There is no formal agency for ethical approval of survey and register-based studies in Denmark. The present study was, however, presented to the regional research scientific ethical committee in Southern Denmark in February of 2017 and deemed not required to obtain formal ethical approval as the study only used questionnaire-based data, according to governing law in Denmark. Information about the survey was provided to potential participants in an invitation letter, and the participants’ voluntary completion and return of the survey questionnaires constituted implied consent. The University of Southern Denmark (SDU) is the controller for processing of personal data in connection with the project, which has been included in SDU’s internal record of processing activities under file number [18/28527], cf. GDPR Article 30.

### Statistical Analysis Plan

All statistical analyses were conducted in SPSS Version 26. The relationships of CU traits to pre-treatment variables were assessed by partial bivariate correlations controlling for pre-treatment within-rater scores on the SDQ externalizing problems scale. This allowed for an assessment of the unique contribution of CU traits, as co-occurring levels of conduct problems and hyperactivity/impulsivity constitute the central aspects of externalizing problems (Lynam et al., 2009).

The association between CU traits and treatment completion was assessed by binary logistic regression of the two non-completion categories combined against the completed category. The conditional predictive value of CU traits on treatment service parameters, e.g., treatment length, were assessed by bivariate partial correlation controlling for baseline parent-rated externalizing problems. The prediction of CU traits on the continuous outcome measures was assessed by multiple regression analyses. As we were interested in the additional predictive value of CU traits, we controlled for baseline externalizing behavior as they might predict treatment gains to a large extent (Hogue et al., 2016). In addition, age, gender and duration of treatment were included in the regression analyses as potential covariates. Possible interaction effects of CU traits and gender on the continuous outcome measures were also tested using the regression analyses.

Given that CU traits are meant to identify a more severe subgroup amongst adolescents with CD (Viding and Kimonis, 2018), a lack of predictive validity of CU traits among adolescents with sub-clinical levels of conduct problems might mask this relationship. We therefore reran all our analyses on the predictive effects of CU traits using the clinically elevated subsample of adolescents that either by self- or parent-report on the SDQ conduct scale scored above the 95%-ile of the Danish norms (Arnfred et al., 2019). Any discrepant results from the analyses of this subsample compared to the full sample are reported.

To test whether CU traits were reduced over time in the context of FFT-treatment, both individual-centered and average-based change statistics were calculated (Estrada et al., 2018). The reliable change index (RCI; Jacobson and Truax, 1991) was used as the individual-centered statistic and computed by dividing ICU12 change scores by the standard error of the difference between the two scores. This allowed a count of the number of cases reliably improving (RCI scores equal or lower than −1.96), reliably worsening (RCI scores equal to or higher than 1.96) and not reliably changing (RCI values between −1.96 and 1.96) over the time of treatment. A repeated measures effect size (*d*_*RM*_) was calculated as the average-based change statistics over the time of treatment (Morris and DeShon, 2002; Lenhard and Lenhard, 2016). Also change statistics were calculated separately for the subgroup of adolescents with elevated levels of CU traits at the start of treatment, using a cut-off of 0.5 *SD* above the mean as done in several previous studies (Viding et al., 2008; van Baardewijk et al., 2009; Andershed et al., 2018; Frogner et al., 2018). We examined the relationship between change in CU traits and change in the continuous outcome measures by correlation analyses.

Although several hypotheses were tested in this study, we chose not to adjust the significance level to reduce the risk of type II errors. As the study was investigating the extent to which a particularly high-risk group of adolescents does not benefit from treatment, and the hypothesized associations were assumed to be correlated, we deemed it more important to keep a standard significance level of 0.05 to be able to identify possible effects, than to risk not detecting these by applying easily available but conservative adjustments of p-values, such as Bonferroni corrections (Bender and Lange, 2001).

As none of the study variables predicted attrition, missing data was assumed to be missing at random (MAR) and handled by pairwise deletion in the correlation and regression analyses. There were 3 outliers in relation to treatment pacing with more than 60 days between sessions, and these values were treated as missing in our analyses.

## Results

### Sample Descriptives

Table 1 presents the descriptive statistics of the main study variables at baseline and post-treatment. There were no significant differences on any of the main study variables a baseline between the 242 study participants who completed questionnaires at both time points and the 165 that only completed at baseline. About one third of the sample (31.2%) scored above the mathematically derived cut-off score of 15 scale points on the ICU12. At baseline a total of 218 adolescents (53.6%) were rated above the 95%-ile on the SDQ conduct problems scale on either self- or parent-report according to Danish norms (Arnfred et al., 2019). The descriptive statistics for this clinically elevated subsample are presented in Table 2.

**TABLE 2:** Descriptive statistics of the main study variables for the clinically elevated subsample.

	Baseline				Post-treatment			
	*n*	*M*	*SD*	Range	*n*	*M*	*SD*	Range
**ICU12**								
Parent-report	218	15.86	6.8	2–33	113	12.12	6.2	1–32
**SDQ-externalizing**								
Parent-report	218	11.71	3.5	2–20	117	8.32	3.8	1–17
Self-report	201	10.06	3.6	2–20	106	9.03	3.7	0–19
**SDQ-emotional problems**								
Parent-report	218	4.88	2.5	0–10	117	3.54	2.4	0–10
Self-report	208	4.33	2.7	0–10	106	3.75	2.6	0–10
**SDQ-prosocial behavior**								
Parent-report	218	6.40	2.1	0–10	117	7.38	2.1	2–10
Self-report	208	7.14	2.1	0–10	106	7.58	1.9	2–10
**SCORE-15**								
Parent-report	217	2.76	0.71	1.0–4.8	115	2.21	0.58	1.1–3.9
Self-report	205	2.89	0.70	1.0–4.7	103	2.41	0.71	1.0–4.3

**ICU, inventory of callous unemotional traits; SCORE, systemic clinical outcome and routine evaluation; SDQ, strength and difficulties questionnaire*.*

### Baseline CU Traits and Problem Severity

The baseline zero-order correlations between CU traits and the measures of adolescent and family functioning and covariates are presented in Table 3 for the full sample and Table 4 for the clinically elevated subsample.

**TABLE 3:** The correlations between the study variables at baseline, gender and age for the full sample.

	1	2	3	4	5	6	7	8	9	10	11
(1) ICU12	–										
(2) SDQ-Ext-P	0.616***	–									
(3) SDQ-Ext-S	0.292***	0.467***	–								
(4) SDQ-Emo-P	–0.058	0.158**	0.061	–							
(5) SDQ-Emo-S	−0.160**	–0.036	0.233***	0.457***	–						
(6) SDQ-Pro-P	−0.642***	−0.427***	−0.179***	0.025	0.088	–					
(7) SDQ-Pro-S	−0.310***	−0.204***	−0.288***	0.005	0.147**	0.381***	–				
(8) SCORE-15-P	0.435***	0.387***	0.181***	0.160**	0.083	−0.285***	−0.130*	–			
(9) SCORE-15-S	0.360***	0.317***	0.369***	0.061	0.197***	−0.258***	−0.293***	0.424***	–		
(10) Gender	0.027	0.141**	0.033	−0.151**	−0.374***	–0.048	−0.148**	–0.009	−0.133**	–	
(11) Age	0.148**	–0.036	0.018	–0.087	0.005	–0.079	0.039	0.047	0.203***	−0.153**	–

*p < 0.05, **p < 0.01, ***p < 0.001, n = 372-407. ICU, inventory of callous unemotional traits; SDQ, strength and difficulties questionnaire; Ext, externalizing; Emo, emotional symptoms; Pro, prosocial scale; SCORE, systemic clinical outcome and routine evaluation; S, self-report; P, parent-report.

**TABLE 4:** The correlations between the study variables at baseline, gender and age for the clinically elevated subsample.

	1.	2.	3.	4.	5.	6.	7.	8.	9.	10.	11.
(1) ICU12	–										
(2) SDQ-Ext-P	0.485***	–									
(3) SDQ-Ext-S	0.049	0.130	–								
(4) SDQ-Emo-P	–0.072	0.159*	0.073	–							
(5) SDQ-Emo-S	−0.236**	–0.120	0.267***	0.517***	–						
(6) SDQ-Pro-P	−0.565***	−0.278***	0.012	0.045	0.157*	–					
(7) SDQ-Pro-S	−0.231**	–0.099	−0.226**	0.044	0.161*	0.297***	–				
(8) SCORE-15-P	0.312***	0.150*	–0.048	0.069	–0.046	–0.119	–0.103	–			
(9) SCORE-15-S	0.232**	0.082	0.161*	0.018	0.160*	–0.096	−0.234**	0.291***	–		
(10) Gender	0.059	0.226**	0.064	−0.234***	−0.415***	–0.093	−0.191**	0.047	–0.122	–	
(11) Age	0.189**	–0.030	0.062	–0.039	0.021	–0.074	0.064	0.013	0.222**	−0.139*	–

*p < 0.05, **p < 0.01, ***p < 0.001, n = 200–218. ICU12, inventory of callous unemotional traits; SDQ, strength and difficulties questionnaire; Ext, externalizing problems; Emo, emotional symptoms; Pro, prosocial behaviors; SCORE, systemic clinical outcome and routine evaluation; S, self-report; P, parent-report.

Table 5 presents the partial correlations between CU traits and the outcome measures when controlling for externalizing problems in both the full and the clinically elevated subsample.

**TABLE 5:** Partial correlations between Callous-Unemotional traits and problems indicators and covariates at baseline.

			SDQ emotional symptoms		SDQ prosocial behavior		SCORE-15	
	Age	Gender	Parent	Self	Parent	Self	Parent	Self
Full sample (*n* = 371–402)	0.214***	–0.067	−0.200***	−0.246***	−0.535***	−0.257***	0.268***	0.302***
Clinically elevated subsample (*n* = 199–216)	0.233**	–0.054	−0.177**	−0.260***	−0.516***	−0.234**	0.277***	0.254***

**p < 0.01, ***p < 0.001. Numbers are partial correlations controlling for within-rater SDQ-externalizing scores. SCORE, systemic clinical outcome and routine evaluation. SDQ, strength and difficulties questionnaire.

### CU Traits and Treatment Outcomes

Among all cases, 268 (65.8%) completed, 47 (11.5%) dropped out, 75 (18.4%) were non-completers and 17 (4.2%) were missing therapist-reported completion data. The results of the binary logistic regression model including both baseline externalizing and baseline CU traits as predictors, showed that CU traits were not significantly related to treatment non-completion, OR: 1.036, 95% CI [0.998, 1.08], *p* = 0.067 in the full sample. In the clinically elevated subsample, CU traits did statistically significant predict treatment non-completion, OR: 1.053, 95% CI [1.003, 1.106], *p* = 0.037. However, the sensitivity of the model in its ability to correctly predict treatment non-completion, was relatively poor, with only 12.2% correct identification of non-completers in the clinically elevated subsample.

Treatment parameters showed that initial treatment pacing between the first two sessions averaged 9.46 days, *SD* = 8.2, the total number of sessions averaged 11.8, *SD* = 5.6, and treatment duration averaged 160.2 days, *SD* = 72.3. The partial correlations of CU traits to treatment parameters controlling for baseline parent-ratings on the SDQ-externalizing scale are presented in Table 6 for both the full sample, and the clinically elevated subsample. No significant findings were observed, apart from the weak negative relation to the number of sessions in the third treatment phase in the clinically elevated subsample, *r*(206) = −0.166, *p* = 0.016.

**TABLE 6:** The partial correlation between the Inventory of Callous-Unemotional traits and treatment parameters when controlling for pre-treatment externalizing scores.

	Days 1st to 2nd session	Days 2nd to 3rd session	Sessions phase 1	Sessions phase 2	Sessions phase 3	Total sessions	Treatment length
Full sample	0.009	−0.013	0.054	−0.016	−0.069	−0.022	0.038
Clinically elevated subsample	0.021	0.075	0.038	−0.080	−0.166*	−0.113	0.011

*p < 0.05, n = 198–389.

The post-treatment improvement ratings averaged 3.39, *SD* = 0.92, for adolescent-report, 3.50, *SD* = 0.89, for parent-report and 3.06, *SD* = 1.08 for therapist-report. Table 7 presents the results of the regression analyses modeling the relationship of baseline CU traits to treatment outcomes, including baseline values of the outcome measure, age, gender and treatment duration as covariates. Table 8 presents the results of the same regression analyses using the clinically elevated subsample only. The interaction term of gender and CU traits was not statistically significant when tested in these regression analyses.

**TABLE 7:** Predictors of treatment outcomes in the full sample.

	*n*	ICU12	Baseline score	Age	Gender	Duration	*R*^2^	*F*
**SDQ-externalizing:**								
Parent	214	0.056	0.601***	–0.057	0.034	0.065	0.417	29.70***
Adolescent	201	0.039	0.696***	−0.113*	−0.120*	–0.052	0.520	42.20***
**SCORE-15:**								
Parent	209	0.082	0.459***	0.030	–0.088	0.151*	0.279	15.75***
Adolescent	196	0.052	0.531***	–0.090	–0.090	–0.057	0.314	17.40***
**Post-treatment improvement rating:**								
Parent	244	−0.212*	0.020^a^	0.088	0.008	0.058	0.045	2.22
Adolescent	224	–0.138	−0.024^a^	–0.003	0.024	0.107	0.034	1.55
Therapist	307	−0.212**	0.077^b^	–0.015	–0.015	0.266***	0.102	6.87***

Table presents regression-based standardized beta coefficients for each predictor. (a) Intra-rater SDQ-Externalizing score used as baseline, (b) Parent-rated SDQ-Externalizing score used as baseline. ICU12, inventory of callous unemotional traits; SCORE, systemic clinical outcome and routine evaluation; SDQ, strength and difficulties questionnaire. *p < 0.05, **p < 0.01, ***p < 0.001.

**TABLE 8:** Predictors of treatment outcomes in clinically elevated subsample.

	*n*	ICU12	Baseline score	Age	Gender	Duration	*R*^2^	*F*
**SDQ-externalizing:**								
Parent	109	0.051	0.402***	–0.057	0.155	–0.007	0.245	6.67***
Adolescent	99	–0.068	0.635***	–0.107	–0.134	–0.045	0.416	13.26***
**SCORE-15:**								
Parent	107	0.097	0.396***	0.071	–0.043	0.114	0.206	5.23***
Adolescent	96	0.068	0.487***	–0.130	–0.062	–0.024	0.252	6.07**
**Post-treatment improvement rating:**								
Parent	123	–0.181	0.052^a^	0.036	–0.004	0.102	0.035	0.85
Adolescent	109	–0.124	−0.013^a^	–0.014	0.042	0.082	0.026	0.55
Therapist	157	−0.239**	0.007^b^	–0.125	–0.023	0.200*	0.135	4.72***

Table presents regression-based standardized beta coefficients for each predictor. *p < 0.05, **p < 0.01, ***p < 0.001, (a) Intra-rater SDQ-externalizing score used as baseline, (b) parent-rated SDQ-Externalizing score used as baseline. ICU12, inventory of Callous Unemotional Traits; SCORE, systemic clinical outcome and routine evaluation; SDQ, strength and difficulties questionnaire. *p < 0.05, **p < 0.01, ***p < 0.001.

To further explore the significant predictions of CU traits on post-treatment improvement ratings, Table 9 presents the results of explorative correlation analyses between baseline CU traits and each item of the measure across responder groups.

**TABLE 9:** Partial correlations of Callous-Unemotional Traits against single item scores on the post-treatment outcome rating across responder groups.

	Post-treatment improvement rating item					
Rater	General	Family communication	Adolescents behavior	Parenting	Parental supervision	Family conflict
Parent	–0.095	−0.143*	−0.161*	–0.094	−0.176**	–0.078
Adolescent	–0.095	–0.040	–0.094	–0.122	−0.185**	–0.098
Therapist	−0.133*	−0.179**	−0.139*	−0.146*	−0.161**	−0.170**

*p < 0.05, **p < 0.01, n = 222–305. Numbers are partial correlations controlling for the duration of treatment and the pre-treatment externalizing score of the respective respondent, with parent scores used as the control variable for the therapist ratings.

### The Malleability of CU Traits

The computation of a reliable change index for CU traits from baseline to post-treatment showed that for the 216 adolescents with available post-treatment CU ratings, 37 (17.1%) were reliably declining, 166 (76.9%) had no reliable change and 13 (6.0%) were reliably increasing. The corresponding repeated measures effect size (*d*_*RM*_) for the group average reduction was 0.36, 95%CI [0.15, 0.53]. The threshold value of elevated CU traits generated as 0.5 *SD* above the mean of the ICU12 was 16 scale points, which coincided well with the mathematically derived cut-off value of 15 scale points. In the subgroup of 60 adolescents with elevated pre-treatment ICU12 scores and an available post-treatment score, 26 (43.3%) were reliably declining, 32 (53.3%) had no reliable change and 2 (3.3%) were reliably increasing, with a corresponding group average based *d*_*RM*_ of 0.91, 95%CI [0.70, 1.46]. Table 10 presents the correlations between change in CU traits and change in the continuous outcome measures.

**TABLE 10:** The correlation between the change scores of the Inventory of Callous-Unemotional traits and the change scores of externalizing behavior and family functioning.

	ΔSDQ-externalizing-P	ΔSDQ-externalizing-Y	ΔSCORE-15-P	ΔSCORE-15-Y
Full sample ΔICU	0.514***	0.113	0.252***	0.151*
Clinically elevated subsample ΔICU	0.507***	0.143	0.231*	0.121

*p < 0.05, ***p < 0.001, n = 94–215. SCORE, systemic clinical outcome and routine evaluation; SDQ, strength and difficulties questionnaire.

## Discussion

This study had three aims focused on increasing our knowledge about the relationship between CU traits and treatment outcomes in the context of FFT, an evidence-based program for adolescent behavior problems. Firstly, we wanted to assess whether CU traits are indicative of more severe behavior problems in a Danish at-risk adolescent population. Secondly, we wanted to investigate the extent to which CU traits at baseline predict treatment outcomes in FFT. Lastly, we wanted to know the proportion of observed reliable change in CU traits among the adolescents that receive FFT-treatment. We used data from an evaluation of FFT in Denmark and the FFT quality assurance database, to study a mixed gender sample of 407 adolescents.

### CU Traits and Problem Severity

The results showed that at baseline, CU traits were associated with higher levels of behavior problems, poorer family functioning, and lower levels of emotional problems and prosocial behavior. This adds to the evidence that among adolescents with behavior problems, CU traits are related to more severe behavior problems, lower levels of anxiety and lower inclination to oblige to social norms (Frick et al., 2014a). The observed lack of gender differences in CU-scores and positive relationship of age to CU, is contrary to previous research (Stickle et al., 2011; Carvalho et al., 2018). This could be due to design and sample variations, as most earlier studies have used longitudinal study designs and population samples, while the current sample is a clinic-referred cross-sectional sample. As CU traits are related to more persistent behavior problems, it is likely that in a clinical sample the proportion adolescents with higher levels of CU traits is larger among the older adolescents. With respect to gender, adolescent boys and girls that are referred to family therapy for behavior problems, might be more similar with respect to CU traits, than what has been observed in other samples.

### CU Traits and Treatment Outcomes

With respect to the question of whether CU traits at baseline were associated with treatment outcomes, our results were somewhat mixed. We did observe a statistically significant result for baseline CU traits to predict treatment non-completion in the clinically elevated subgroup. However, the specificity of the prediction was very low, making it hard to argue that this finding is clinically relevant. Furthermore, baseline CU traits did not predict the number of days between the three initial sessions, the number of treatment sessions nor the overall duration of treatment. Only in the clinically elevated group did baseline CU traits show a weak negative correlation to the number of sessions in the last treatment phase, generalization. This implies that adolescents with a combination of severe conduct problems and CU traits could be more likely to drop out during or before this last treatment phase. As a result, they might receive fewer sessions focused on maintaining use of new skills, relapse prevention and addressing risk factors outside the family system, which are the aims of the final treatment phase in FFT (Alexander et al., 2013).

The general reductions from baseline to post-treatment for both externalizing behavior and family problems has been reported for the larger sample from which data for this study was drawn (Vardanian et al., 2019). The current results indicate that CU traits at baseline do not impede the observed changes in parent- and adolescents-reported outcomes. Across raters, the baseline scores of both externalizing behavior and family functioning were consistently the strongest statistically significant predictor of post-treatment score, and CU traits were not predictive of these outcomes. This was also the case in the clinically elevated subsample. In line with the findings by White et al. (2013), this suggests that treatment outcomes in FFT might be less impacted by CU traits compared to what has been observed in studies of other treatments including adolescent aged participants (e.g., Falkenbach et al., 2003; Spain et al., 2004; Masi et al., 2013).

In relation to the post-treatment improvement ratings, baseline CU traits were negatively related to parent- and therapist-report, but not self-report. Similar findings were also observed by White et al. (2013) using the same post-treatment questionnaire and could suggest that CU traits are linked to diminished therapeutic gain in areas that aren’t captured by the SDQ and SCORE-15 measures. Exploratory analyses of each item on the improvement rating scale, showed that CU traits were related to lower improvement ratings of parental supervision across all rater groups. This could suggest that there are challenges related to parental supervision of adolescents with CU traits, that are not sufficiently addressed by FFT treatment. One such challenge might be the fact that adolescents with CU traits are likely to be more deceitful and strategically misreport with respect to their whereabouts, activities and social contacts. This might lower the effectiveness of FFT interventions related to improving parent-youth communication and relationships, family contracting and family problem solving. Further investigation into this area is important as poor parental supervision has been linked to increased adolescent behavior problems and a high-persistent CU-trajectory (Waller et al., 2018a).

Additionally, the item-by-item analyses showed that baseline CU traits were related to lower reports of change on adolescent behavior and family communication from both parents and therapists. This contrasts the findings related to change in externalizing behavior and family communication as measured by the SDQ and the SCORE-15, respectively. One possible explanation for this could be that the improvement rating scale is more representative of a general sense of treatment satisfaction, which has demonstrated a negative relationship to baseline CU traits (Bjørnebekk and Kjøbli, 2017). Lower treatment satisfaction among therapists and parents might well be expected as adolescents with CU traits will have higher levels of problems post-treatment, despite obtaining similar or even greater reductions during FFT treatment (White et al., 2013). This points to the need for future work to develop interventions that are even more appropriate, tailored and effective for adolescents with CU traits. They might be in need of even more effective treatment options in order to have their behavior problems reduced sufficiently and sustainably to be comparable to adolescents without such traits.

### The Malleability of CU Traits

Lastly, this study examined the level of change in CU traits occurring over the course of FFT treatment. The majority (76.9%) of the study participants had no reliable change, as hypothesized, while 17.1% had a reliable decline and 6.0% had a reliable increase. The theoretical values of standardized reliable change scores beyond −1.96 and +1.96 within a normal distribution is 2.5% in each direction. This means that in the whole sample we observed more cases both reliably declining and reliably increasing than what would be expected by measurement error and chance. These proportions were markedly different in the subsample with elevated levels of CU traits at baseline, where 43.3% had a reliable decline, 53.3% had no reliable change and 3.3% had a reliable increase. This represents a much larger proportion of cases reliably declining and a similar proportion of cases reliably increasing, compared to what would be expected by chance. This therefore provides some support for the therapeutic malleability of CU traits in adolescence.

The increased proportion of reliable reductions in CU traits observed for the high CU group could entail that baseline CU traits act as a moderator for the malleability of CU traits in the context of FFT. For those with higher levels of CU traits at the start of treatment, we are more likely to observe a reliable decline during treatment. This is clinically relevant, as one might argue that only those with higher scores are in need of reducing their CU traits. This potential moderating effect of baseline CU might explain the somewhat mixed results from previous research (Wilkinson et al., 2016). In addition, one should be aware that most research on the malleability of CU in adolescents, including our own, have studied interventions that were not necessarily directly targeting CU. Given that CU traits have been shown to be relatively stable during adolescence (López-Romero et al., 2014), the possibility of change during treatments not specifically targeting these traits might be limited. With this in mind, the results from this study suggests that at least some elements of the FFT program might be relevant when designing interventions aimed at reducing CU traits.

Changes in CU traits were positively correlated with changes in externalizing problems and family functioning as reported by parents. This indicates that, from a parental perspective, changes in CU traits coincide closely with changes in externalizing behavior and to some extent with changes in family functioning. By youth-report, however, there was only a weak positive correlation between changes in CU trait and the change in family functioning. This could be due to the lower level of cross-rater correlations between CU traits and externalizing behavior as demonstrated in the baseline correlations and other studies (Docherty et al., 2016; Thøgersen et al., 2020). The fact that change in youth-reported family functioning showed a relationship to change in parent-reported CU traits, might be indicative of the importance of parental warmth as a potential protective factor in the development of CU traits (Kroneman et al., 2011; Pasalich et al., 2012; Fanti et al., 2017; Waller et al., 2018b; Ray et al., 2019). It could also suggest that if treatment can help parents to see their youth with more potential for empathy and prosocial behavior, the youth will experience an improvement in family level functioning. Future research is needed to shed more light on the directionality, strength and mechanisms by which CU traits interact with various aspects of the parent-youth relationship and family level functioning.

### Limitations

This study has its strengths in investigating the association between CU traits and treatment outcomes in a large clinical sample of adolescents with behavior problems that received a well-established evidence-based treatment. There are, however, some limitations of the study pertinent to the interpretation of its findings. In the first place, this study used a single group pre-post-treatment design, which limits causal inferences. Secondly, a good proportion of the sample did not complete the study questionnaires at post-treatment, which lowers the study’s statistical power and generalizability. Thirdly, this study assessed the affective dimension of psychopathy, but not the other dimensions of psychopathy which could have provided improved predictions (Andershed et al., 2018). Fourthly, trauma history was not assessed and analyses could not be conducted separately for possible primary and secondary CU variants that might differ in how they influence outcomes (Craig and Moretti, 2018). Fifthly, we did not include baseline therapist-report of externalizing problems and used parental-report as a substitute in the analysis related to therapist-reported outcomes. Lastly, in this study, CU traits was measured by parent-report only, which have shown low inter-rater reliability toward self-report (Thøgersen et al., 2020), and single rater data potentially increases the risk of assessment bias (Gao and Zhang, 2015; Hemmingsson et al., 2016). Self-reported CU traits might have served as a better predictor of treatment outcomes and be less susceptible to change (Butler et al., 2011).

### Future Directions

The results of this study indicate that CU traits do not seem to impede treatment completion or improvements in externalizing behavior and family functioning observed in FFT treatment. However, a relationship between CU traits and lower improvements in parental supervision and a lower number of generalization sessions was observed. This suggests that for adolescents with CU traits these treatment areas might need additional interventions and tailoring, than what is already embedded within the FFT program as implemented at these Danish sites. With respect to the malleability of CU, this study demonstrated that a good portion of adolescents with high levels of CU traits at baseline gained reliable reductions in CU traits. Changes in CU traits were furthermore related to changes in externalizing behavior and family functioning.

These findings raise several questions and ideas for future research. First of all, further research is needed to determine whether CU traits predict diminished improvements in parental supervision in other contexts, and identify interventions that might be more suited to increase parental supervision for adolescents with CU traits. Second, the potential long-term predictive effect of CU traits on treatment improvements should be studied, to see if CU traits can indicate reduced longevity of treatment results or increased long-term risk of relapse. Third, more research is needed to understand the therapeutic processes and interventions that can result in attenuation of CU traits. Although plausible explanations could be given for FFT (motivation strategies, strength-based interventions and increases in parental warmth), as has been given for a mental models approach (positive emotions and prosocial strategies; Salekin et al., 2012), the mechanisms of these change processes are not yet established. Furthermore, we should aim to increase our knowledge on how and to what extent a reduction of CU traits should be a prioritized focus in the treatment of adolescents with CD and CU traits. Given the multiple risk-factors that are often present in these adolescents’ lives, therapists are in need of guidance on the degree to which and when treatment should intervene specifically on CU trait reduction compared to prioritizing goals and tailoring interventions related to other well-established risk factors for adolescent behavior problems (e.g., negative parenting, family conflict, dysfunctional communication, and negative peer influence).

## Data Availability Statement

The datasets generated for this study will not be made publicly available because: the data contains sensitive data related to personal mental health information that is considered pseudonymized data by the legal advisory board at the University of Southern Denmark. Requests to access the main dataset can be directed to CS, css@vive.dk. Requests to access the quality assurance dataset can be directed to DT, d.m.thogersen@nubu.no.

## Ethics Statement

Ethical review and approval was not required for this study in accordance with the local legislation and institutional requirements. Written informed consent from the participants’ legal guardian/next of kin was not required to participate in this study in accordance with the national legislation and the institutional requirements. Participants received information about the survey in an invitation letter and voluntary completion and return of the survey questionnaires constituted an implied consent.

## Author Contributions

CS, DT, ME, and GB contributed to the conception and design of the study. CS and DT organized the database. DT performed the statistical analysis with guidance from ME and GB. DT wrote the first draft of the manuscript. All authors contributed to manuscript revision, read, and approved the submitted version.

## Conflict of Interest

DT is occasionally hired to provide training, supervision, and consultation in the Functional Family Therapy model in Denmark, which could be construed as a potential conflict of interest. The remaining authors declare that the research was conducted in the absence of any commercial or financial relationships that could be construed as a potential conflict of interest.
